# HSV-1 Triggers an Antiviral Transcriptional Response during Viral Replication That Is Completely Abrogated in PKR^−/−^ Cells

**DOI:** 10.3390/pathogens12091126

**Published:** 2023-09-03

**Authors:** Rosamaria Pennisi, Maria Teresa Sciortino

**Affiliations:** Department of Chemical, Biological, Pharmaceutical and Environmental Sciences, University of Messina, Viale Ferdinando Stagno d’Alcontres 31, 98166 Messina, Italy

**Keywords:** HSV-1, innate antiviral response, PKR

## Abstract

The activation of the innate immune response during HSV-1 infection stimulates several transcription factors, such as NF-κB and IRF3, which are critical regulators of IFN-β expression. The released IFN-β activates the ISGs, which encode antiviral effectors such as the PKR. We found that HSV-1 triggers an antiviral transcriptional response during viral replication by activating TBK1-IRF3-NF-κB network kinetically. In contrast, we reported that infected PKR^−/−^ cells fail to activate the transcription of TBK1. Downstream, TBK1 was unable to activate the transcription of IRF3 and NF-κB. These data suggested that in PKR^−/−^ cells, HSV-1 replication counteracts TBK1-IRF3-NF-κB network. In this scenario, a combined approach of gene knockout and gene silencing was used to determine how the lack of PKR facilitates HSV-1 replication. We reported that in HEp-2-infected cells, PKR can influence the TBK1-IRF3-NF-κB network, consequently interfering with viral replication. Otherwise, an abrogated PKR-mediated signaling sustains the HSV-1 replication. Our result allows us to add additional information on the complex HSV-host interaction network by reinforcing the concept of the PKR role in the innate response-related networks during HSV replication in an in vitro model.

## 1. Introduction

Efficient HSV-1 replication depends on the delicate balance between host immune surveillance and the production of viral proteins, which occurs with an accurate gene transcription program. All HSV-1 genes are expressed sequentially, starting with the immediate early (IE) gene group (ICP0, ICP4, ICP22, ICP27, and ICP47) responsible for gene regulation; followed by the early (E) gene group, involved in the replication of the viral DNA genome; and finally the late (L) group, including structural proteins required for the progeny viruses [1,2,3,4]. Once HSV enters, it activates multiple transduction signals, including nuclear factor kappa B (NF-κB), IFN regulatory factors (IRFs), and mitogen-activated protein kinase (MAPK) pathways, resulting in the induction and production of cytokines, IFNs, and chemokines in infected cells [5,6]. Cytokines and chemokines regulated in HSV-infected cells limit replication and the spread toward neighboring cells by recruiting the innate immune cells (macrophages, NK, T cells) to the site of HSV infection [7]. HSV-1 encode viral proteins, which affect multiple steps of innate immune signaling. Indeed, HSV-1 manipulates the cytosolic pattern recognition receptors which are essential sensors of viral DNA and stimulate the interferon-genes-mediated antiviral immunity. Upon viral infection, pattern-recognition receptors (PRRs) recognize various pathogen-associated molecular patterns (PAMPs) [8,9]. HSV-1 activates either PRRs, which usually sense DNA nucleic acids, such as TLR9, IFI16, DAI, and AIM2, then TLR3, TLR7, TLR8, RIG-I, and MDA5, which are activated by RNA nucleic acids, or viral infection-associated cellular double-stranded RNA (dsRNA) [10]. PRRs activate downstream adapters such as STING that recruit TBK1 and IKK kinase complex to activate IRF3 and NF-kB [11,12]. Phosphorylated IRF3 translocates to the nucleus and induces the expression of type I IFNs, which exert an autocrine and paracrine effect inducing an antiviral state in infected cells and neighboring non-infected cells [13]. Among the molecules with critical biological functions induced by IFN is PKR [14,15,16,17]. PKR provokes a global shutdown of protein translation by blocking eIF2α [18,19,20]. Induction of the IFN cascade during viral infection is crucial for activating innate host defense [21]. In this scenario, the capability to recognize viral nucleic acids in the host cell cytoplasm is a critical step. In addition to its translation regulation function, the PKR protein is also involved in the signal transduction processes and transcription control mediated by the NF-κB pathway and in other biological effects such as differentiation and autophagy, cell death, and inflammasome activation and apoptosis [22,23,24,25,26]. PKR has been demonstrated to interact directly with the C-terminal part of p53 and phosphorylates p53 on the Ser392 residue. Once activated, PKR supports a transcriptional response involved in cell cycle arrest or apoptosis [27]. The coordinated recruitment of host transcription factors affects the virus replication and minimizes the viral cells’ spread. Thus, HSV-1 uses its viral proteins to counteract the innate antiviral mechanisms by targeting the STING pathway [28,29]. Here, we verified the impact of PKR in the innate response network by genome editing and gene silencing approaches. Analyses of viral genes and the active replication of HSV-1 were performed in PKR knocked-out and parental cell lines to examine whether the loss of PKR protein, involved in the innate immune response, rescues the viral replication. Similarly, we depleted PKR by transfecting cells with siRNAs specifically directed against PKR and monitored the change in viral gene expression after transfection. Our results demonstrate that HSV-1 triggers an antiviral transcriptional response in HEp-2 cells completely abrogated in PKR^−/−^ cells. The deficiency in PKR expression and its functionality facilitates active HSV-1 replication. Our study provides a better understanding of the complex HSV-host interaction network and remarks on the central role of PKR in cellular antiviral defense.

## 2. Materials and Methods

### 2.1. Cell Culture

Cell lines were originally obtained from the American Type Culture Collection (ATCC). Vero cell lines were propagated in Eagle’s Minimum Essential Medium (EMEM, Lonza, Belgium), supplemented with 6% fetal bovine serum (FBS) (Euroclone), 100 U/mL penicillin and 100 μg/mL streptomycin mixture (Lonza, Belgium). HEp-2 cells (human larynx epidermoid carcinoma cell lines) and PKR-deficient HEp-2 cells (PKR^−/−^), kindly provided by Professor Zhou G. (Shenzhen International Institute for Biomedical Research, Shenzhen, Guangdong, China), were grown in Roswell Park Memorial Institute (RPMI) 1640 medium (Lonza, Belgium) and supplemented with 10% of FBS, 100 U/mL penicillin, and 100 μg/mL streptomycin mixture. All cell lines were grown at 37 °C in a 5% CO_2_ incubator.

### 2.2. Virus and Viral Infections

HSV-1 strain F is a prototype HSV-1 strain kindly provided by Professor Bernard Roizman (University of Chicago, Chicago, IL, USA). The experimental infections were carried out by exposure of all cell lines to the HSV-1 virus, strain F, at a multiplicity of infection (MOI) of 10, according to the experimental design. The adsorption of the virus was carried out for 1 h at 37 °C with gentle shaking. After infection, the supernatant was replaced with a fresh culture medium. Then the infected cultures and related controls were incubated at 37 °C, under 5% CO_2_, and collected at the established times of the experimental design. Immunoblot analysis of lysates of PKR-deficient HEp-2 cells (PKR^−/−^) and wild-type HEp-2 was performed to evaluate the knockdown of PKR. The details of the western blot analysis and the PKR and GAPDH antibodies are given below.

### 2.3. Design of PKR siRNAs and Detection of the Transcript Levels in HEp-2 Cells

The gene sequence of Homo sapiens eukaryotic translation initiation factor 2 alpha kinase 2 (EIF2AK2), transcript variant 1, was obtained from the NCBI website with accession number NM_002759.3. Specific sequences of PKR siRNAs (si-PKR#1, si-PKR#2, si-PKR#3) and scrambled siRNAs (with no complementary target sequence in the human genome) were reported in Table 1 and were synthesized by Genepharma (Suzhou, China). To determine the efficiency of PKR suppression, western blot analysis was performed by transfecting siRNAs at 5 and 10 µM for 48 h as reported in the 2.4 paragrapher. All transfections were carried out using Lipofectamine™ RNAiMAX Transfection Reagent (Invitrogen, Waltham, MA, USA) according to its manufacturer’s instructions, and PKR silencing was validated by Real-Time PCR. The transfection was performed by using si-PKR#2 for 48 h, and then the samples were collected and subjected to total RNA extraction as reported in the 2.6 paragrapher. The purified PCR product was loaded on the 1.5% agarose gel. After electrophoresis, the expected PKR band was detected in wild-type HEp-2 cells but not in cells following si-PKR#2 silencing.

### 2.4. Western Blot Analysis

Immunoblot analysis was carried out to evaluate the accumulation of PKR protein. The procedure was published previously [31]. Briefly, total cells lysates were prepared from cells by SDS sample buffer 1X [62.5 mM Tris-HCl (Tris(hydroxymethyl)aminomethane hydrochloride) pH 6.8; 50 mM DTT (dithio-threitol); 10% glycerol; 2% SDS (sodium dodecyl sulfate); 0.01% Bromophenol Blue; EDTA-free Protease Inhibitor Cocktail 1X (Roche, Basel, Switzerland)], sonicated, and boiled for 5 min. An equal amount of protein extracts was subjected to SDS-gel electrophoresis (SDS-PAGE), transferred to membranes (Bio-Rad Life Science Research, Hercules, CA, USA), and subjected to immunoblot analysis. Specific bands were visualized using Immobilon Classico Western HRP substrate (Merk, Millipore, Rahway, NJ, USA). Anti-GAPDH (sc-32233) and PKR (A12) (sc393038) were purchased from Santa Cruz Biotechnology (Santa Cruz, CA, USA), and secondary HRP-conjugated goat anti-rabbit IgG was purchased from Millipore.

### 2.5. DNA Extraction

The viral DNA was extracted with phenol/chloroform solution and precipitated from the organic phase. The procedures were published previously [32]. The DNA pellet was washed twice in a solution containing 0.1 M trisodium citrate in 10% ethanol and dissolved in 8 mM NaOH. The DNA concentration was determined by fluorometer analysis with the Qubit double-stranded DNA (dsDNA) HS (High Sensitivity) Assay Kit according to the manufacturer’s instructions. Viral DNA amplification was carried out using TaqMan™ Universal Master Mix II (Applied Biosystems™, Foster City, CA, USA) in a 50 µL reaction mixture containing TaqMan Universal Master Mix II, DNA (100 ng), HSV-1 forward (10 µM), and HSV-1 reverse (10 µM) primers, TaqMan probe (5 µM). The sequence of primers is shown in Table 2. The amplification was carried out on Applied Biosystems 7300 Real-Time PCR System under the following conditions: 10 min at 95 °C, 60 s at 95 °C for 40 cycles, 30 s at 60 °C, and 30 s at 72 °C. Absolute quantification Real-time PCR using a specific TaqMan probe was performed to detect viral DNA. Viral load was derived from the threshold cycle (CT) using the standard curve generated in parallel, and the result is expressed as a concentration in µg of DNA/µL.

### 2.6. RNA Extraction, Reverse Transcription, and Real-Time PCR

Total RNA was extracted using TRIzol^®^ (Life Technologies, Carlsbad, USA), according to the manufacturer’s instructions, and DNase-treated before cDNA transcription as follows: 1 μg of RNA was incubated at 37 °C for 2 h with 5 μL 10X DNase I Buffer, 2 μL Recombinant RNase-free DNase I (10U) (2270A TaKaRa, Dalian, China) and RNase inhibitor (20U) (N251A Promega). The procedures were published previously [32]. Total RNA (1 μg) was reverse transcribed using ReverTra Ace^®^ qPCR RT Master Mix (FSQ-201 Toyobo) under the following conditions: 37 °C for 15 min, followed by 50 °C for 5 min and 98 °C for 5 min. The cDNAs were used for quantitative Real-Time PCR carried out on Applied Biosystems 7300 Real-Time PCR System. The thermal profile consists of a 10 min incubation at 95 °C followed by 30 cycles of 15 s denaturation at 95 °C, 35 s annealing at 60 °C and 45 s elongation at 72 °C. The cDNA copy numbers were normalized to GAPDH. The analytic primers for RT-PCR are reported in Table 2. Each quantitative Real-time PCR experiment includes a minus-reverse transcriptase control.

### 2.7. Statistical Analysis

Three independent experiments in triplicate (n = 3) were carried out for each assay, and the results represent the average ± standard deviation (SD). Statistical analysis was performed with GraphPad Prism 8 software (Graph-Pad Software, San Diego, CA, USA) using one-way variance analysis (ANOVA). The significance of the *p*-value was indicated with asterisks (**, ***, ****), which indicate the significance of the *p*-value less than 0.01, 0.001, and 0.0001, respectively.

## 3. Results

### 3.1. Impact of PKR Knockout in the TBK1-IRF3-NF-κB Network

**Figure 1:**
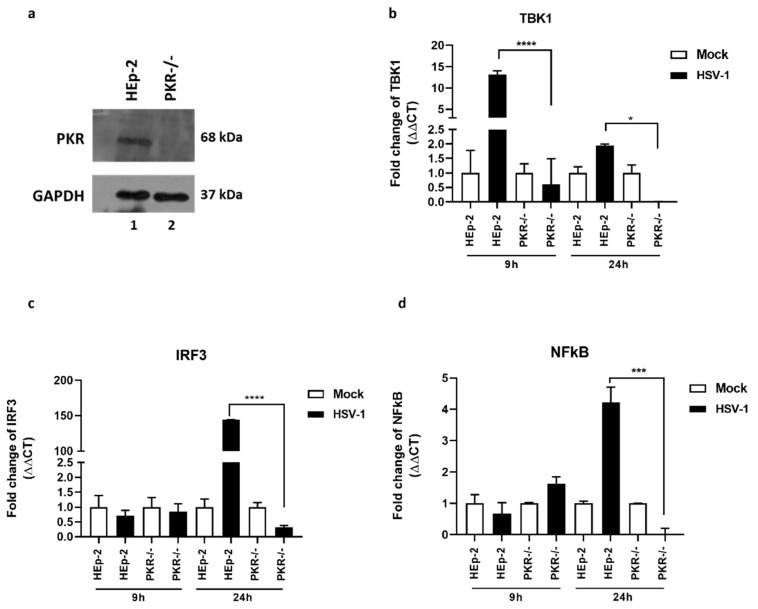
Abrogated activation of TBK1, IRF3 and NF-κB during HSV-1 replication in PKR^−/−^ cells. (**a**) Immunoblot analyses of lysates of PKR^−/−^ and wild-type HEp-2. (**b**–**d**) Quantitative Real-time PCR was performed to evaluate the transcriptional levels of TBK1 (**b**), IRF3 (**c**), and NF-κB (**d**) in HEp-2 and PKR^−/−^ cells. The cells were infected at MOI 10 with HSV-1 and harvested at 9 h and 24 h post-infection to total RNA extraction. The transcriptional levels of considered genes were analyzed by calculating the value of 2^−ΔΔCt^. The assay was performed as a means of triplicate ± SD and expressed as fold change over the housekeeping genes. **** <0.0001; *** <0.001; * <0.01.

The role of PKR in the antiviral response is often associated with the inhibition of translation of both viral and cellular mRNA due to the inactivation of eIF2α factor [18,19,20]. The PKR-kinase activity is involved in cellular growth regulation, inflammation, cancer, and neurodegenerative processes by acting on the activation of intracellular signals such as autophagy and apoptosis [21,22,23,24,25,26,27]. The activation of the antiviral immune response exerted by PKR downstream-sensing viral nucleic acid stimulates and coordinates the expression of the innate antiviral response [33]. In this scenario, the TBK1-IRF3-NF-κB network plays a central role in the antiviral immune response. Therefore, these experiments aimed to evaluate the impact of PKR protein on the transcription levels of the TBK1-IRF3-NF-κB network during HSV-1 replication. Thus, we used PKR^−/−^ validated by immunoblot (Figure 1a). HEp-2 and PKR^−/−^ cells were infected or mock-infected with HSV-1 and collected at 9 h and 24 h. The comparison of the transcriptional levels of TBK1, IRF3, and NF-κB was reported in Figure 1. We found that upon HSV-1 infection, the transcripts levels of TBK1 peak at 9 h post-infection in HEp-2 wt but not in PKR^−/−^ cells (Figure 1b). Downstream from the TBK1 upregulation, at 24 h we found the accumulation of IRF3 (Figure 1c) and NF-κB (Figure 1d) transcripts in HSV-1 infected cells. In contrast, we reported that HSV-1 infected PKR^−/−^ cells fail to upregulate TBK1. Downstream from TBK1 activation, HSV-1 fail to upregulate IRF3 and NF-κB. These data suggested that PKR exert a crucial role in the TBK1-IRF3-NF-κB network during HSV-1 replication.

### 3.2. Depletion of PKR Uniquely Improves Viral DNA Accumulation and HSV-1 Gene Expression

**Figure 2:**
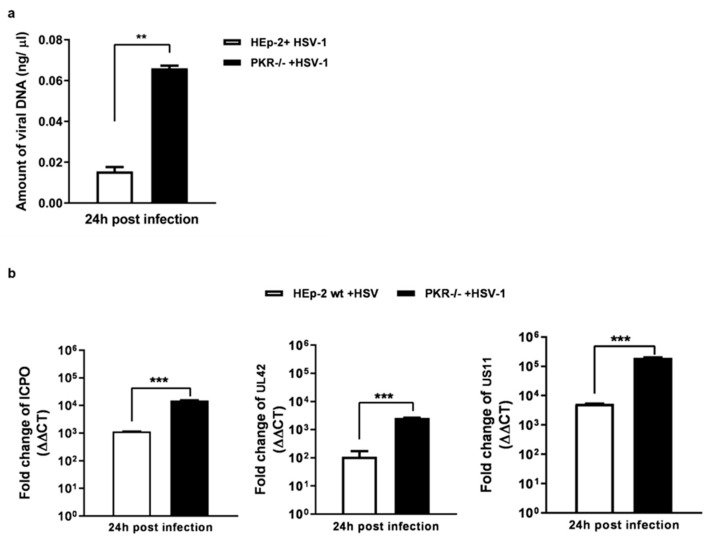
Analysis of viral DNA and representative viral transcripts in PKR^−/−^ compared to HEp-2 cell lines. PKR^−/−^ and parental cell lines were infected with HSV-1 at MOI 10 and collected 24 h post-infection. (**a**) The absolute quantification of Real-time PCR was used to detect the amount of viral DNA in both cell lines by using a specific TaqMan probe. Viral load was derived from the threshold cycle (CT) using the standard curve generated in parallel, and the result is expressed as a concentration in ng of DNA/μL. (**b**) Expression of viral transcripts cascades in HEp-2 and PKR^−/−^ cell lines. The mRNA was purified with Trizol according to the manufacturer’s instructions. The measure of changes in the expression level of ICPO, UL42, and US11 genes was analyzed by calculating the value of 2^−ΔΔCt^. The assay was performed as a means of triplicate ± SD and expressed as fold change over the housekeeping genes. *** < *p* < 0.0001 and ** <0.01.

Although PKR has a role in the innate immune reaction against viruses, its contribution during HSV replication is still unclear. To add new information on its potential role, we compared the viral replication efficiency in PKR^−/−^ cells and HEp-2, quantifying the viral DNA (Figure 2a) and the viral transcripts, such as ICPO, UL42, and US11 (Figure 2b). The absolute quantification of Real-time PCR was used to measure the amount of viral DNA in PKR^−/−^ and HEp-2 cells. As shown in Figure 2a, the amount of viral DNA significantly (*** *p* < 0.001) increases in PKR^−/−^ compared to HEp-2 cells at 24 h post-infection. To determine whether the higher accumulation of viral-DNA correlated with the changes in the viral genes’ expression, we analyzed ICP0, UL42, and US11 viral transcripts accumulation as representative genes of a sequential genetic cascade of HSV-1. Figure 2b demonstrates significantly (*p* < 0.0001) higher levels of ICPO, UL42, and US11 in PKR^−/−^ cells than in HEp-2 parental cell lines, highlighting that the lack of viral control mediated by PKR allows the virus to improve its replicative capability. These findings suggest that the inactivation of PKR fully rescues the HSV-1 replication.

### 3.3. Effect of PKR-Silencing during HSV-1 Replication

**Figure 3:**
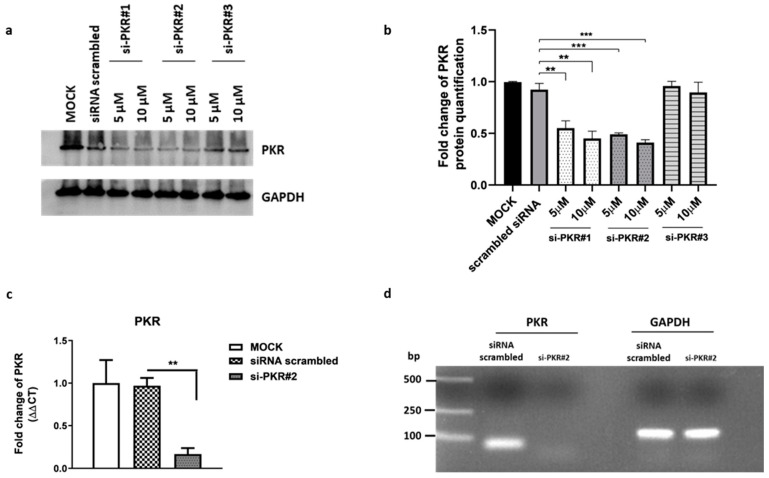
Validation of PKR silencing by western blotting and RT-qPCR. HEp-2 cells were transfected with three different siRNA duplexes at 5 and 10 µM to silencing PKR, and siRNA scrambled was used as negative control (10 µM). Samples were collected at 48 h post-transfection to measure the expression of PKR. (**a**) Western blot analysis was performed to detect the protein level of PKR following silencing. The GAPDH was used to check the protein loading. Panel (**b**) shows fold changes in protein quantification. Band density was determined with the Image J program, expressed as fold change over the appropriate housekeeping genes and with respect to siRNA scrambled control. The data were graphically represented by using GraphPad Prism 6 software. (**c**) Quantitative Real-time PCR was performed to evaluate the transcriptional levels of PKR in HEp-2 transfected with si-PKR#2 (10 µM) and siRNA scrambled. (**d**) The amount of PKR transcripts obtained following transfection with si-PKR#2 was confirmed by running the amplicons on an agarose gel. Statistical analyses were performed with one-way analysis of variance (ANOVA) in triplicate, and asterisks (** and ***) indicate the significance of *p*-values less than 0.01 and 0.001, respectively.

**Figure 4:**
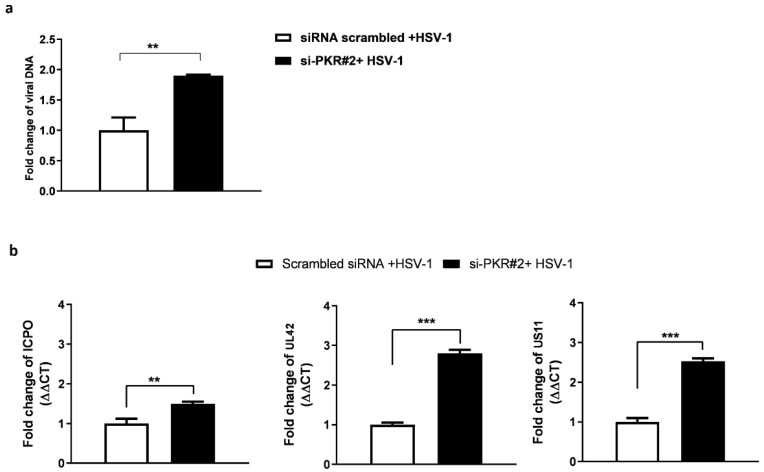
Accumulation of HSV-1 DNA and representative viral transcripts following PKR silencing. HEp-2 cells were transfected with si-PKR#2 (10 µM) and infected with HSV-1 (10 MOI). Scrambled siRNA was used as no-target control. The samples were collected 24 h post-infection (**a**) viral DNA, and (**b**) viral transcripts were analyzed. The changes levels of ICPO, UL42, and US11 genes were analyzed by calculating the value of 2^−ΔΔCt^. The assay was performed as means of triplicate ± SD and expressed as fold change over the housekeeping genes. *** < *p* <0.0001 and ** <0.01.

To verify whether the observed effects on virus replication were specific to the knockdown of PKR and not due to artificial off-target effects, we planned a series of experiments by silencing the PKR gene. First, PKR siRNAs duplex were designed to knock down PKR gene expression in HEp-2 cells. The transfection was carried out by using three siRNAs whose sequences are reported in Table 1. The si-PKR#1 was previously reported [34], and it was used in this study as a PKR silencing positive control. The si-PKR#2 and si-PKR#3 were designed by the PKR sequence template (accession number NM_002759.3). As shown by immunoblot analysis, si-PKR#1 and si-PKR#2 efficiently knocked down total PKR protein accumulation in HEp-2 cells (Figure 3a), unlike si-PKR#3. Therefore, the duplex si-PKR#2 was used for further experiments, and PKR mRNA levels were analyzed by real-time RT-PCR (Figure 3b,c). To determine whether PKR depletion affected the replication of HSV-1, HEp-2 cells were transfected with si-PKR#2 for 24 h, infected with HSV-1 (MOI 10), and collected 24 h post-infection to evaluate the viral DNA and viral genes transcriptional levels by real-time PCR (Figure 4). The results report that following PKR silencing, the replication of HSV-1 in HEp-2 cells was favored. A substantial increase in viral DNA synthesis (Figure 4a) and viral gene accumulation (Figure 4b) was detected in si-PKR#2 silenced infected cells compared to nontargeting siRNA control. These results confirm that the recovery of viral replication observed in PKR^−/−^ cells was due to the specific knockdown of PKR and its key role in the antiviral response to HSV-1.

## 4. Discussion

The successful host response to viral infections depends on intracellular signaling pathways triggered downstream by virus-induced initial activation of PRRs. Thus, DNA and RNA viruses stimulate in the cell host the expression of pro-inflammatory cytokines or recruiting of immune cells and release of IFNs which establish an antiviral state [34]. Induction of IFNs cascade during viral infections is a crucial step in triggering the innate host defense. IFNs cascade is regulated by important transcription mediators among these the IRFs, which contain a conserved interferon consensus sequence (ICS) binding region, and TBK1 [35]. TBK1 activates downstream IRF3 and subsequent IFN-I production, which interacts with the universally expressed interferon receptor (IFNAR) that allows the expression of ISGs [12]. PKR is an ISG product with kinase activity that recognizes and binds dsRNA sequences on conserved double-stranded RNA binding domains (dsRBDs) in its N-terminal domain [36]. Viruses characterized by an RNA genome, the dsRNAs, responsible for PKR activation, represented the replicative forms and intermediates for the genomic RNA synthesis [16]. In viruses with a complex DNA genome characterized by open reading frames in opposite orientations, such as HSV, the PKR activation in infected cells can be triggered by the mRNA transcripts overlapping [31,37]. The binding of dsRNA to the dsRBDs causes a conformational change in PKR towards an active form which exposes the catalytic site for autophosphorylation and the phosphorylation of eIF-2α at residue S51 resulting in the shutdown of all translation of 5′capped mRNA [18,19,20,38]. Thus, PKR activation prevents the synthesis of viral proteins. In addition, to its translational regulatory function, PKR was reported to promote the NF-κB activation via phosphorylation of IkB and subsequently promote the RLR-mediated type I interferon signaling [39,40]. PKR is also required to activate inflammasomes, subsequently release proinflammatory cytokine HMGB1 [41], and induce apoptosis process [42]. During viral infection, several viruses synthesize viral proteins that modulate key regulators of cellular events and influence them to their own benefit [43]. The NF-κB pathway controls several intracellular events and, by contrast, is a target of human viruses with a direct mechanism or with the involvement of upstream regulators. HIV activates NF-kappaB via physical interaction with IκB-α and p65 [44]; HSV-1 activates NF-κB early by the binding of the gD envelope glycoprotein to the herpesvirus entry mediator A (HveA) [45] and by PKR [31]; and Hepatitis B and Papillomaviruses target cellular regulator proteins, which normally instigate NF-κB activation [46,47]. Similarly, IRF pathway members are also targets of viral evasion. Indeed, while the US3 protein of HSV hyperphosphorylates and inhibits IRF3 to block IFNβ production [45], the C6 protein of vaccinia virus binds to the TBK1 adaptor proteins and prevents IRF3 activation, and UL46 of HSV impairs the interaction of TBK1 and IRF3 and downregulates the activation of IRF3 [48,49]. TBK1 plays a central role in coordinating the innate immune response to cytosolic nucleic acids, which are detected by cytosolic nucleic acid sensors [12,36,50]. In activating the antiviral immune response, PKR senses endogenous viral nucleic acid and stimulates and coordinates the expression of innate antiviral response. Thus, our purpose was to verify the impact of PKR in the TBK1-IRF3-NF-κB network at transcriptional levels during HSV replication. We report that PKR knockdown cells were defective in recruiting TBK1 and, therefore, unable to activate IRF3 and NF-κB (Figure 1). Indeed, our results showed that PKR was required for TBK1 induction in response to infection by HSV. The transcriptional levels of TBK1 are stimulated by viral infection at 9 h post-infection and subsequently allow the accumulation of IRF3 and NF-κB transcripts. This event matches with the accumulation of PKR transcripts which, during HSV-1 replication, peaks at 9 h post-infection [31], and demonstrated that HSV-1 triggered host cell sensors leading to activation of IRF3 and NF-κB signaling in a PKR-dependent manner. Abrogated accumulation of TBK1, IRF3, and NF-κB transcripts was found during HSV-1 replication in the absence of the PKR. Thus, to verify whether the lack of PKR was advantageous for HSV, we detected viral DNA and viral gene transcripts in PKR^−/−^ cells (Figure 2). We showed that PKR^−/−^ cells were resistant to HSV-1 replication compared to parental cells. A similar result was obtained by specifically silencing PKR (Figure 4). These data add new information to the data reported in the literature, where it has been demonstrated that TBK1 is essential for interferon-beta (IFN-beta) production and innate antiviral immunity. In turn, TBK1 is important for both IRF3 and NF-κB activation. NF-κB synergizes with IRF3 to induce high levels of type I IFN and other proinflammatory cytokines [12,51]. In conclusion, the results reported here demonstrate that PKR is essential for antiviral signaling mediated by TBK1-IRF3-NF-κB networks during HSV-1 replication. In particular, the lack of PKR facilitates viral replication, impacting the innate cellular response.

## Figures and Tables

**Table 1:** si-PKRs oligo sequences.

Direction	si-PKR#1 (nt. 584–604) *	si-PKR#2 (nt. 786–806)	si-PKR#3 (nt. 639–658)
TARGET	GAGAATTTCCAGAAGGTGA	TTCAGGACCTCCACATGATAG	TTCAGGACCTCCACATGATAG
SENSE	GAGAAUUUCCAGAAGGUGA	UUCAGGACCUCCACAUGAUAG	UUCAGGACCUCCACAUGAUAG
ANTISENSE	UCACCUUCUGGAAAUUCUC	CUAUCAUGUGGAGGUCCUGAA	CUAUCAUGUGGAGGUCCUGAA
siRNA-Scrambled			
SENSE			UUCUCCGAACGUGUCACGUTT
ANTISENSE			ACGUGACACGUUCGGAGAATT

* From Watanabe T. et al., 2013 [30].

**Table 2:** Sequences of primers used to measure gene expression.

Gene	Forward	Reverse
ICP0	5′-TCTGCATCCCGTGCATGAAAAC-3′	5′-CTGATTGCCCGTCCAGATAAAG-3′
UL42	5′-CTCCCTCCTGAGCGTGTTTC-3′	5′-CACAAAGCTCGTCAGTTCGC-3′
US11	5′-GGCTTCAGATGGCTTCGAG-3′	5′-GGGCGACCCAGATGTTTAC-3′
TBK1	5′-GCAGTTTGTTTCTCTGTATGGC-3′	5′-AATGTTACCCCAATGCTCCA-3′
IRF3	5′-GCCGAGGCCACTGGTGCATAT-3′	5′-TGGGTCGTGAGGGTCCTTGCT-3′
NF-ΚB	5′-TAAAGCCCCCAATGCATCCAAC-3′	5′-CCAAATCCTTCCCAGACTCCAC-3′
GAPDH	5′-GAGAAGGCTGGGGCTCAT-3′	5′-TGCTGATGATCTTGAGGCTG-3′
PKR	5′-CAGGCAAACAAGGTCCCATC-3′	5′-GCGAGTGTGCTGGTCACTAA-3′
HSV-1	5′-CATCACCGACCCGGAGAGGGAC-3′	5′-GGGCCAGGCGCTTGTTGGTGTA-3′
HSV-1 TaqMan probe		5′-6FAM-ccgccgaactgagcagacacccgcgc-TAMRA ^1^

^1^ 6FAM: 6-carboxyfluorescein and TAMRA is 6-carboxytetramethylrhodamine.

## Data Availability

The data presented in this study are available on request from the corresponding author.
